# Prenatal and postnatal diagnosis of congenital hymen prolapse: A case report

**DOI:** 10.1097/MD.0000000000035700

**Published:** 2023-10-27

**Authors:** Maochun Zhang, Xueying Wang, Jiao Chen, Shichun Wang, Menglin Luo

**Affiliations:** a Department of Obstetrics and Gynecology, Affiliated Hospital of North Sichuan Medical College, Nanchong, Sichuan, China; b Department of Ultrasound, Affiliated Hospital of North Sichuan Medical College, Nanchong, Sichuan, China.

**Keywords:** case report, hymen, prolapse, ultrasound diagnosis

## Abstract

**Rationale:**

Female genital tract abnormalities are relatively uncommon and usually discovered accidentally. And hymen prolapse is even rarer, which is asymptomatic and is commonly found during the examination of the baby external genitals.

**Patient concerns:**

Here, we report a case of fetal genital abnormality detected at 32 weeks of gestation.

**Diagnoses:**

At 32^+1^ weeks of pregnancy, ultrasound showed taht an iso-echoic mass of about 8 mm × 5 mm was protruding from the genitalia, and at 36 weeks, ultrasound showed that an iso-echoic mass of about 9 mm × 5 mm could be seen protruding from the genitalia, and its morphology was similar to the result of the 32-week data. At 39 weeks a baby girl was naturally delivered. Physical examination showed the female external genitalia, part of the hymen protruded into the vaginal orifice. Finally, the clinical diagnosis was hymen prolapse.

**Interventions and outcomes:**

No treatment was carried out. Reexamination at 11 days after birth revealed a significantly smaller prolapse than before. Since the postpartum follow-up, the baby has been in good condition, the hymen has gradually returned, and the genitals are all normal.

**Lessons:**

Regardless of the confidentiality of prenatal tests regarding the sex of the fetus, prenatal ultrasound should be used to fully evaluate the morphology and structure of each system including the reproductive system of the fetus when screening fetal malformations. The purpose of this case is to remind doctors of the rigorous degree of genital examination, increase the detection rate, and save the life of the fetus.

## 1. Introduction

Female genital tract abnormalities are relatively uncommon and are usually discovered accidentally. These types of abnormalities are usually asymptomatic and do not require special treatment. The hymen is a type of connective tissue membrane in the perineum, which closes the vaginal orifice. Hymen atresia is a condition in which the vagina is abnormally closed or absent. It usually occurs in infancy at an incidence of 0.014%~0.100%,^[1]^ causing vaginal hemorrhage and fluid accumulation. Hymen prolapse is asymptomatic and is commonly found during the examination of the baby external genitals. So far, only a few studies reported on hymen prolapse. Here we reported a single case of congenital hymen prolapse in a baby girl, which was discovered by ultrasound of the fetus and consequently confirmed after the baby was born.

## 2. Case presentation

**Figure 1.**
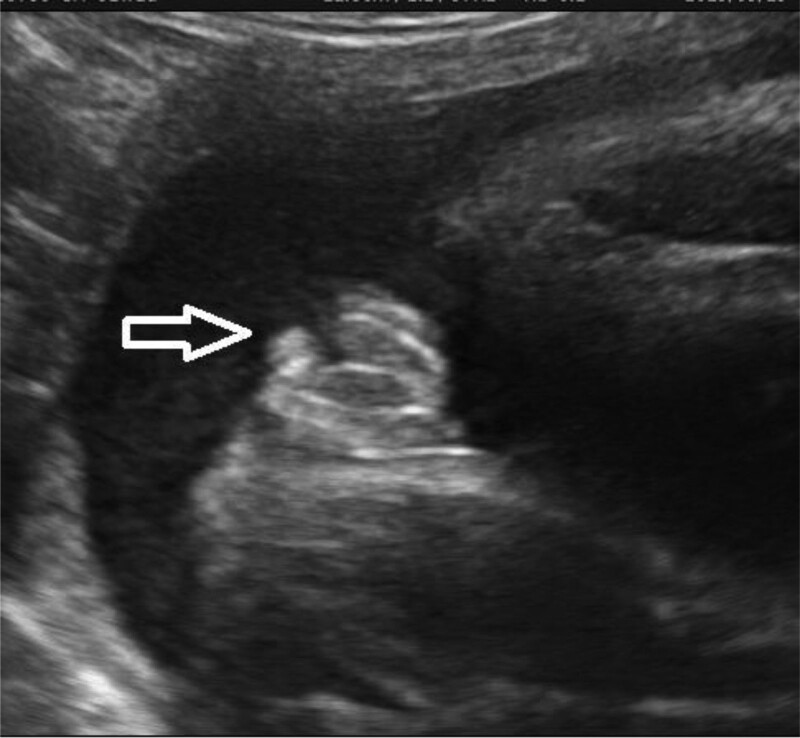
Ultrasound examination of the fetus. At 32^+1^ wk of pregnancy, ultrasound showed the female genitalia, the labia were visible, and an iso-echoic mass of about 8 mm × 5 mm was protruding from the genitalia.

A 36 years old pregnant woman, G1P0, at 32^+1^ weeks of pregnancy underwent a routine fetal ultrasound examination in our hospital. Ultrasound showed the female genitalia, the labia were visible, and an iso-echoic mass of about 8 mm × 5 mm was protruding from the genitalia (Fig. 1). The woman and her husband were in good health and had no genetic disease history. This was a natural pregnancy, and there was no history of special medication. The 13-week and 24-week ultrasound examinations suggested that fetal growth and development were normal, and there were no obvious abnormalities. The patient received a prenatal diagnosis at 24 weeks due to her advanced age. The result of amniocentesis showed 46, XX, and no obvious chromosomal structural abnormalities. Moreover, the fetal size, placental thickness, and amniotic fluid depth were all within the normal range; the fetus kidneys were normal, and the bladder was visible. The anal “target-ring sign” was also visible.

At 36 weeks of pregnancy, color ultrasound examination of the pregnant woman 1 month later showed that fetal size, placental thickness, and amniotic fluid depth were all within the normal range, and no obvious abnormalities were observed in the fetal urinary system. An iso-echoic mass of about 9 mm × 5 mm could be seen protruding from the genitalia, and its morphology was similar to the result of the 32-week data.

**Figure 2.**
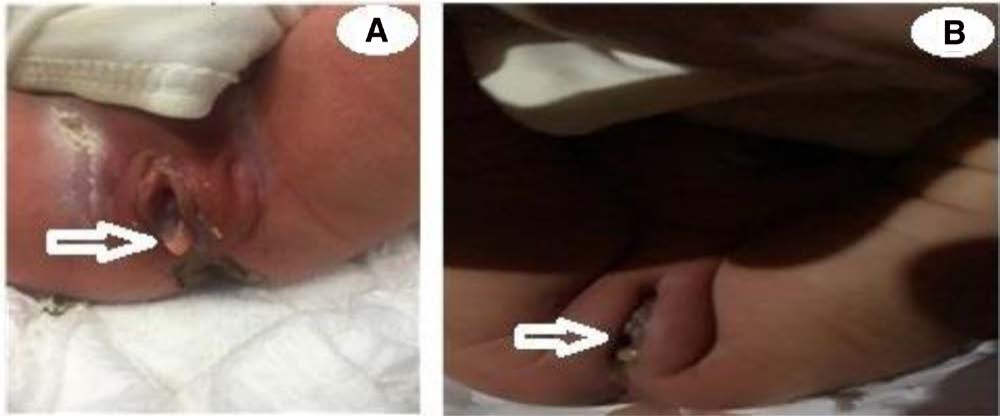
Physical examination. (A) Physical examination showed the female external genitalia, part of the hymen protruded into the vaginal orifice, a needle-like hole was visible in the hymen, and the urethra and anal opening were normal. (B) Reexamination at 11 d after birth revealed a significantly smaller prolapse than before.

At 39 weeks of pregnancy, the pregnant woman was admitted to the hospital due to vaginal bleeding for 1 hour, and a baby girl was naturally delivered. The Apgar score for 1 minute, 5 minutes, and 10 minutes was 10 points. Physical examination showed the female external genitalia, part of the hymen protruded into the vaginal orifice (Fig. 2A), a needle-like hole was visible in the hymen, and the urethra and anal opening were normal. Therefore, the clinical diagnosis was hymen prolapse.

Reexamination at 11 days after birth (Fig. 2B) revealed a significantly smaller prolapse than before. During this time, the parents did not complain that the baby girl was particularly unwell. The previous diagnosis was further confirmed by follow-up.

## 3. Discussion

The hymen is a remnant of the juvenile state and is a horizontal mucosal fold on the bottom wall at the junction of the urogenital vestibule and the vagina.^[2]^ It usually lasts until the first sexual life. It is formed around the 5^th^ month of fetal development. Yet, its origin is still controversial. The development of the hymen might originate from both the vaginal bulb and the urogenital sinus or from the urogenital sinus alone.^[3]^ Hymen prolapse refers to hyperplasia and hypertrophy of the fibrous connective tissue of the hymen prolapsing outside the vaginal orifice. Herein, we reported a single case of hymen prolapse, which was discovered by ultrasound of the fetus using an ultrasound image that showed vegetations protruding between the labia on both sides.

Thus far, the pathogenesis of the congenital malformation of the female reproductive system has been only investigated in animal models. It is believed that high maternal estrogen levels lead to fetal hymen growth.^[4]^ After birth, the fetus is no longer exposed to the high estrogen environment of the mother, and in the new environment, which is more similar to menopause situation regarding estrogen levels; the enlarged hymen gradually shrinks and retracts into the vagina. For infant hymen hyperplasia and prolapse, special treatment is generally not required. If urination and defecation are affected, partial hymen resection could be performed under local anesthesia.

The common causes of abnormal external genitalia in fetuses include ambiguous external genitalia gender, hypospadias, clitoris hypertrophy, and hymen polyps. For fetuses with ambiguous external genitalia gender, chromosome examination could be performed to genetically determine the fetus gender. Ultrasound follow-up could then be used to determine the condition of the genitals, and if necessary, fetoscopy could be performed. Hypospadias mainly occurs in male fetuses and has an incidence of 0.02%~0.41%.^[5]^ The sonographic characteristics of fetal hypospadias mainly include^[6,7]^: the end of the penis becomes blunt; the penis is bent to the ventral side; the penis is short and small; urinating from an abnormal opening on the ventral side of the penis rather than from the tip of the penis; the flow of urine is not straight but fan-shaped; the dorsal foreskin of the penis is thickened and looks like a turban; The common feature of severe hypospadias fetuses is the “tulip” sign formed by the separation of the short and bent penis and scrotum.

In this case, the fetus was genetically identified as a female baby, and 2 parallel lines of external genitalia were observed on the ultrasound image. Clitoral hypertrophy refers to the length of the clitoris exceeding 1 cm.^[8]^ Anatomically, the clitoris is located closer to the urethra; an enlarged clitoris is generally located in front of the genitals. At the same time, the prolapsed hymen might be closer to the anus. These 2 abnormalities can be easily distinguished after the fetus was born, and usually do not affect the child life. Hymen polyps refer to the elongated protrusions of hymen tissue, protruding from the edge of the hymen or extending from the edge of the hymen itself, usually <5 mm.^[9]^ It usually protrudes from the lower part (the dorsal side) of the hymen to the outside of the vagina, and most commonly disappears on its own without special treatment,^[10]^ which was difficult to differentiate from hymen prolapse before birth.

In conclusion, prenatal ultrasound should be used to fully evaluate the morphology and structure of each system of the fetus when screening fetal malformations. When necessary, chromosome karyotype analysis, endocrine hormone detection and fetoscope examination should be combined to timely provide pregnant women with correct diagnostic information and guide clinical prognosis.

## Author contributions

**Conceptualization:** Maochun Zhang, Jiao Chen.

**Data curation:** Maochun Zhang, Xueying Wang, Jiao Chen, Shichun Wang, Menglin Luo.

**Funding acquisition:** Maochun Zhang.

**Investigation:** Maochun Zhang, Xueying Wang, Jiao Chen, Shichun Wang, Menglin Luo.

**Resources:** Maochun Zhang.

**Supervision:** Maochun Zhang, Xueying Wang.

**Validation:** Xueying Wang.

**Writing – original draft:** Xueying Wang.

**Writing – review & editing:** Maochun Zhang, Xueying Wang.
